# Characterization of covalent crosslinking strategies for synthesizing DNA-based bioconjugates

**DOI:** 10.1186/s13036-019-0191-2

**Published:** 2019-07-10

**Authors:** Malithi P. Wickramathilaka, Bernard Y. Tao

**Affiliations:** Department of Agricultural and Biological Engineering, Purdue University, 225 S. University Street, 745 Agricultural Mall Drive, West Lafayette, IN 47906 USA

**Keywords:** EDC crosslinking, MALDI-TOF, RP-HPLC, Product yield, Reaction mechanism

## Abstract

An adapted strategy from the conventional 1-ethyl-3-(3-dimethylaminopropyl) carbodiimide hydrochloride (EDC) crosslinking method was developed to form a covalently coupled phosphoramidated single stranded DNA (ssDNA). Matrix assisted laser desorption ionization-time of flight (MALDI-TOF) results demonstrated that the phosphoramidated ssDNA conjugate is stable for several days, and that phosphoramidation occurred exclusively at the 5′ phosphate of ssDNA. A reversed phase high-performance liquid chromatography (RP-HPLC) method with UV detection was developed to determine the yield of conjugates. The methods coefficients of variation (%CV) were less than 6%, and biases ranged from − 5.1 – 1.2%. The conjugate yield via the conventional EDC method was 68.3 ± 2.2%, while that of the adapted EDC/Imidazole method was 79.0 ± 2.4% (*n* = 10). This study demonstrates a convenient one pot strategy for crosslinking biological molecules.

**Electronic supplementary material:**

The online version of this article (10.1186/s13036-019-0191-2) contains supplementary material, which is available to authorized users.

## Introduction

1-Ethyl-3-(3-Dimethylaminopropyl) Carbodiimide (EDC) is a water soluble zero-length crosslinker which is convenient to use and is relatively inexpensive. It has been used in a variety of conjugation techniques to couple carboxyl groups to primary amines [1–3]. EDC first forms an active ester intermediate which then undergoes nucleophilic substitution in the presence of a strong nucleophile, such as a primary amine. EDC crosslinking is generally carried out in buffers devoid of extraneous carboxyls or primary amines at physiological pH. Although EDC crosslinking chemistry is well known, reversion reactions can limit its utility [4, 5]. The O-acylisourea product intermediate can be easily hydrolyzed, reverting to the original carboxylate molecule [1]. To overcome this limitation, sulfo N-Hydroxysuccinimide (sulfo-NHS ester) has been used to form a more stable second intermediate prior to amination [5]. This enhanced EDC/NHS method has been used in a wide range of applications such as immobilizing carboxymethylated coatings on enzyme-linked immunosorbent assay wells [6], immobilizing proteins onto grafted solid surfaces [7, 8], crosslinking antibodies to functionalized solid supports [9], peptide functionalized gold nanoparticles [10], antibody functionalized superparamagnetic nanoparticles [11], grafting polymers onto porous silicon [12], constructing DNA-cellulose nanocrystals [13], and creating nucleic acid functionalized carbon nanotubes [14]. Although EDC and EDC/NHS reactions have been extensively used before, their reaction efficiencies have only been quantified indirectly [9] or in semiquantative FTIR analyses [15].

In the final step of the EDC/NHS reaction mechanism, amination is favored at pH 7.5–8. Therefore, when many researchers used the EDC/NHS method, they have approached a two-step protocol [1, 4, 16]. The initial EDC/NHS reaction is performed at pH 4.5–7.5. Immediately afterwards, the pH of the reaction mixture is raised to approx. 8.0 prior to adding the amine reactant. This is because amination is better facilitated at higher pH. This two-step process faces the risk of hydrolyzing the NHS intermediate, as it has a half-life ranging from 10 min – 1 h [16]. Not only is this two-step process cumbersome, it can also be prone to poor product yields during reactions with enzymes or immunoglobins due to loss of activity. That is because these biomolecules generally have an isoelectric point (pI) around 7.4. Furthermore, sulfo-NHS ester is expensive, and is highly water labile.

In the current study, a novel phosphoramidated single stranded DNA (ssDNA) conjugate was synthesized via a convenient, inexpensive and time efficient one-pot reaction method. An ssDNA modality was chosen to address unique problems related to DNA and nucleic acid bioconjugation techniques. The largest setback of using EDC in nucleic acid studies is due to the formation of non-specific, electrostatic interactions with the phosphate backbone, and open nucleotide base pairs [1, 17]. Additionally, EDC crosslinking can also face problems due to self-dimerization of DNA, preventing covalent bond formation between the desired functional groups. Previous literature does not suggest practical approaches to circumvent such adduct formation, and hence developing alternate protocols to address these drawbacks is important. Thereby, upon identifying the tedious multi-step reactions involved with EDC and EDC/NHS crosslinking, the expenses related to it, and the problematic non-specific binding with DNA, we investigated an alternate route to address these drawbacks.

**Fig. 1:**
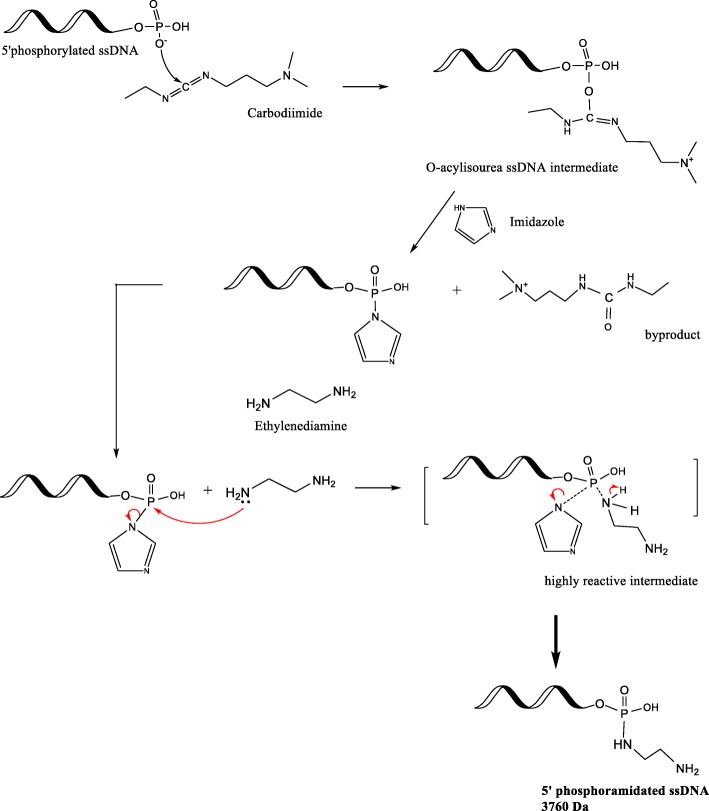
Schematic of the EDC/Im reaction (modified from [1]). The imidazole (at 6.0 pH) and ethylenediamine addition steps are performed within a few seconds in order to drive the forward amination reaction

This study reports using imidazole (Im) to increase the reaction efficiency. The possibility of adding an alkaloid to drive the forward reaction has been previously proposed and tested in binding DNA to microwells [18], and in immobilizing phosphoproteins onto collagen [19]. However, to our knowledge, there are no studies which have reported product characterization, product stability, and product quantitation obtained via the EDC/Im reaction strategy. This study quantitatively compares the conventional EDC and the modified EDC/Im method to crosslink negatively charged functional groups with primary amines in a one-pot reaction scheme, without adjusting the temperature or pH (Fig. 1). This study also reports a chromatographic analytical method suitable to assess DNA conjugates.

The theoretical reaction mechanism is depicted in Fig. 1. At neutral pH, O^−^ in the free 5’phosphate group of the ssDNA attacks the C^δ+^ on the EDC molecule, which then forms an unstable isourea intermediate. Imidazole (Im) at pH 6.0 ensures that one N atom in the ring is always protonated, and enables the other N atom to carry a lone pair of electrons which makes the molecule a strong nucleophile [20]. When imidazole is immediately introduced to the isourea intermediate, the P^δ+^ undergoes nucleophilic substitution by imidazole. The phosphorylimidazole intermediate is highly reactive and will undergo a nucleophilic attack by the primary amine in ethylenediamine (EDA) in an S_N_2 reaction, resulting in a stable, covalent, phosphoramidate bond.

## Materials and methods

### Materials

5’phos- TGT GCA TTA TTT-3′; T_m_ 29.0 °C; molecular weight, 3721 Da, was purchased by IDT® DNA. The sequence was designed with reduced G, C nucleotides, and a medium length in order to avoid secondary structure formation, and for definite molecular weight difference observations after conjugation. Imidazole (Im), EDC and 99% v/v ethylenediamine (EDA) were purchased from Thermo Fisher Scientific. 10 mM sodium phosphate (NaH_2_PO_4_·H2O), 0.15 M NaCl, buffer medium was freshly prepared, pH adjusted to 7.2 ± 0.2, and filtered. 0.1 M Imidazole solutions were prepared and pH adjusted to 6.0 ± 0.2.

### Phosphoramidation of ssDNA; EDC, and EDC/Im

5′ phosphorylated ssDNA as received was dissolved in 50 μL of PBS (pH 7.2 ± 0.2), and was added to EDC (carbodiimide) at a 150-fold molar excess amount to the ssDNA. In order to reduce non-specific EDC binding to the ssDNA phosphate backbone, the molar ratio of EDC: 5′ phosphorylated ssDNA was reduced to 150-fold excess, instead of the 430-fold molar excess suggested in literature [16]. Next, 0.025 M EDA dissolved in PBS (10 mM, pH 6.0 ± 0.2) was added in an equimolar amount to the ssDNA. In order to allow only one covalent bond per one phosphoryl group, the molar ratio of 5′ phosphorylated ssDNA: EDA was maintained at 1:1. The reaction mixture was gently vortexed, and an additional 120 μL of PBS was added to the vial prior to incubation at r.t. (room temperature) for approx. 4 h. In the EDC/Im reaction, instead of PBS, Im (0.1 M, pH 6.0 ± 0.2) was used to dissolve EDA in an equimolar amount to the ssDNA. The molar ratio of 5′ phosphorylated ssDNA: EDA was maintained at 1:1. The reaction mixture was gently vortexed, and an additional 120 μL of Im was added to the vial prior to incubation at r.t. (room temperature) for approx. 4 h. The phosphate concentration of the PBS buffer was limited to 10 mM so that the free phosphate groups did not bind with EDA.

After the 4 h incubation period, each sample was subjected to dialysis against deionized water for 2 h using 2000 MWCO (molecular weight cut-off) dialysis tubes to remove unreacted EDA, Im, and buffer salts.

### Matrix-assisted laser desorption/ionization time-of-flight (MALDI-TOF) analysis

MALDI-TOF spectrometric results were obtained using an Applied Biosystems (Framingham, MA) Voyager DE PRO mass spectrometer. This instrument utilizes a nitrogen laser (337 nm UV laser) for ionization, with a time-of-flight mass analyzer. Prior to MALDI-TOF, each sample was purified using a C18 – ZipTip column (Millipore Corporation, Billerica, MA). The ZipTip columns were conditioned using two aliquots of 10 μL acetonitrile followed by two aliquots of 10 μL 0.1% trifluroroacetic acid (TFA). As ssDNA contains functional groups which readily lose a proton, negative linear mode was used (MH^−^) to produce singly charged species.

### Confirming phosphoramidation exclusively at the 5’phosphate of ssDNA

Since ssDNA is comprised of a phosphate backbone, any charged counter ions or species could interact with these groups, giving rise to weak, unstable electrostatic interactions. To investigate whether EDA is covalently bound exclusively at the 5′ phosphate group of the ssDNA, an EDC/Im reaction with nonphosphorylated ssDNA (3641.4 Da) was conducted. In phosphorylated ssDNA, there is a phosphate group at the end of the strand, while in the nonphosphorylated ssDNA, it is an OH group. As nonphosphorylated ssDNA lacks an open phosphate group susceptible for conjugation, it was not expected to observe a mass signal in the MALDI-TOF spectra at the conjugate mass region.

### Stability of phosphoramidate bond

To investigate the phosphoramidate bond over a prolonged time, MALDI-TOF analyses of the purified products was conducted after 7 days, and 14 days (*n* = 4). These subsequent results for time point 0 (1 day), time point 1 (7 day), and time point 2 (14 day) were obtained from the same product sample which were stored at − 20 °C.

### IP-RP-HPLC conditions

A C18 XTerra® (Waters, MA) column with 4.6 × 100 mm, 3.5 μm particle diameter, and a guard column were used for reversed phase separation and purification of the conjugates. High-performance liquid chromatography (HPLC) was performed on an Alliance 2695 separation module, using Empower version 2.0 software (Waters Corp., Milford, MA), equipped with a Waters 2496 UV/Vis detector (Waters Corp., Milford, MA). Detection was carried out at λ_260 nm_. HPLC analysis was conducted in 0.1 M TEAA, pH 9.70 ± 0.05 (triethylammonium acetate) buffer with 5% acetonitrile (ACN) and 30% ACN as the two mobile phases. 0.1 M TEAA buffer was prepared by adding 5.6 mL of glacier acetic acid to 950 mL of deionized water. To this, 13.8 mL of TEA was added gradually, and the solution was stirred. Afterwards, the pH of the solution was adjusted to 9.70 ± 0.05, and the final volume was brought to 1000 mL by adding water. This solution was filtered using gravity filtration and qualitative-grade filter paper, and was stored at 4 °C. Mobile phases were prepared as follows; A: 5% acetonitrile in TEAA (v/v), B: 30% acetonitrile in TEAA (v/v). The gradient method incorporated was 90–60% A in 16 min, and 60–90% A in the next 14 min. A flow rate of 1.0 mL/min, a sample injection volume of 100 μL, a sample temperature of 33 °C, and a column temperature of 38 °C, were used. The column was equilibrated for 50 mins prior to running samples, and for 30 mins in between different sample injections. The samples were diluted 50% (v/v) in TEAA buffer prior to HPLC experimentation. Each sample was filtered using a 0.45 μm PTFE syringe filter directly into the HPLC vial.

### HPLC: Standards preparation and calibration curve

In order to validate the HPLC method, a calibration curve was generated using four concentration values of the starting material (5’phosphorylated 12 bp ssDNA) in triplicates. 0.4 mg of 5′ phosphorylated ssDNA as received was dissolved in 1 mL of TEAA buffer, and was used as the stock solution, from which the successive aliquots were used for the standards preparation. The dilutions yielded 0.57 mg, 0.285 mg, 0.1425 and 0.07125 mg/mL standard solutions respectively. Each level of concentration was run in triplicates to obtain a total of 12 determinations in the specified range. The precision of an analytical procedure entails the closeness of agreement between a series of measurements obtained by multiple sampling of a homogenous sample. This was assessed by the coefficient of variation (%CV) which was calculated by the following equation: %CV = (standard deviation of the triplicates/mean) × 100. The accuracy of the method was assessed by the %bias from the theoretical concentration, which was calculated by the following equation: % bias = [(calculated concentration – nominal concentration)/nominal concentration] × 100.

Linearity is the procedure’s ability to obtain test results which are directly proportional to the concentration of the standard (5′ phosphorylated ssDNA), and was assessed by fitting the data into a linear regression numerical model. The limit of detection (LOD) of a method is the lowest amount of analyte which can be reliably detected by the method, and was calculated according to the following equation: LOD = (3.3 × σ)/slope. Limit of quantitation (LOQ) is the lowest amount of analyte which can be quantitatively determined with suitable precision and accuracy, and was determined by the following equation: LOQ = (10 × σ)/slope. In both the LOD and LOQ equations, slope = slope of the average regression line. σ is the standard deviation of the y-intercepts of the three separate regression lines. Linearity, LOD, and LOQ were assessed according to the ICH Harmonized Tripartite Guidelines.

### HPLC: Product fraction collection and purification

Conjugate fractions were collected from several injections of the same product sample. The collected fractions were left open overnight in the fume hood to evaporate the organics. The sample was then subjected to dialysis against deionized water for 2 h using 2000 MWCO dialysis tubes. Prior to usage, dialysis tubes were prewashed in deionized water thoroughly to remove any glycerin present in the membranes. The recovered samples were freeze dried at − 80 °C overnight and lyophilized the next day. The conjugate masses were characterized on MALDI-TOF. In order to calculate the amount of conjugate product formed, the area underneath the HPLC chromatograph peaks and the regression equation were utilized. The percentage yield of the conjugate is reported as; [ssDNA conjugate product (mg)/starting ssDNA (mg)] × 100.

## Results and discussion

### Phosphoramidated ssDNA conjugate analysis; MALDI-TOF

**Fig. 2:**
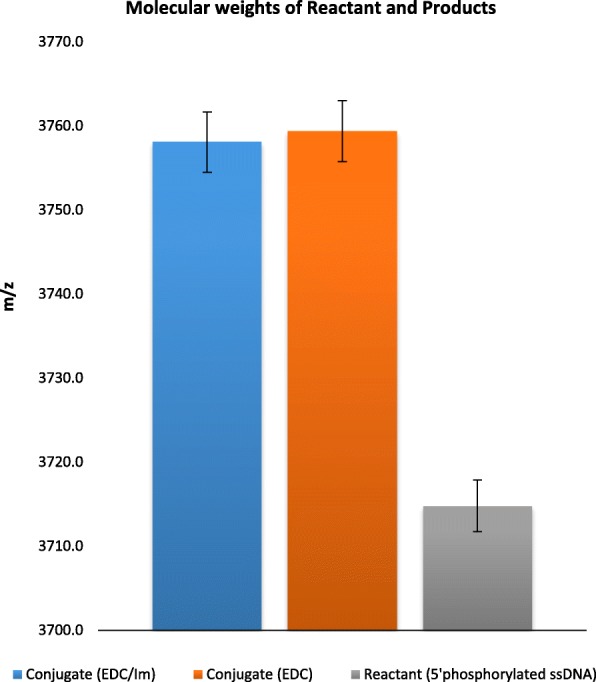
The observed molecular weights of the reactant and conjugates formed via the EDC and EDC/Im reaction methods. A molecular weight difference between the reactant and conjugates are observed. Error bars are represented by ±1 SD

Conjugates formed via the conventional EDC, and the EDC/Im reaction mechanisms were characterized by MALDI-TOF. The expected conjugate molecular weight of 3758 Da was calculated by adding the individual molecular weights of ssDNA and EDA, and subtracting 17 Da to account for the loss of an -OH during conjugation. Figure 2 illustrate that the observed phosphoramidated ssDNA conjugate molecular weight was 3758.5 ± 3.6 Da for the EDC/Im mechanism (*n* = 14) and 3758.4 ± 3.6 for the EDC mechanism (*n* = 7). The average differences between the expected and observed conjugate molecular weights via the EDC/Im and EDC reaction mechanisms were 1.2 Da and 3.3 Da respectively, indicating successful conjugation. The EDC/Im reaction scheme was tested using 15 bp 5′ phosphorylated ssDNA, and MALDI-TOF results indicated successful and repeatable phosphoramidation of 15 bp ssDNA (*n* = 5). The expected molecular weight was 4703.1 ± 4.3, and the observed conjugate molecular weight was 4709.7 ± 3.5 Da Table 1.

**Table 1:** The calculated and experimentally observed (MALDI-TOF) molecular weights

Reactant or product	Average molecular weight (Da)	
	EDC/Im	EDC
12 bp ssDNA (reactant)	3714.9 ± 3.1	3714.4 ± 3.1
Expected conjugate (product)	3757.9 ± 3.1	3757.4 ± 3.1
Observed conjugate (product)	3758.5 ± 3.6	3758.4 ± 3.6

### Stability of phosphoramidated ssDNA conjugates

**Fig. 3:**
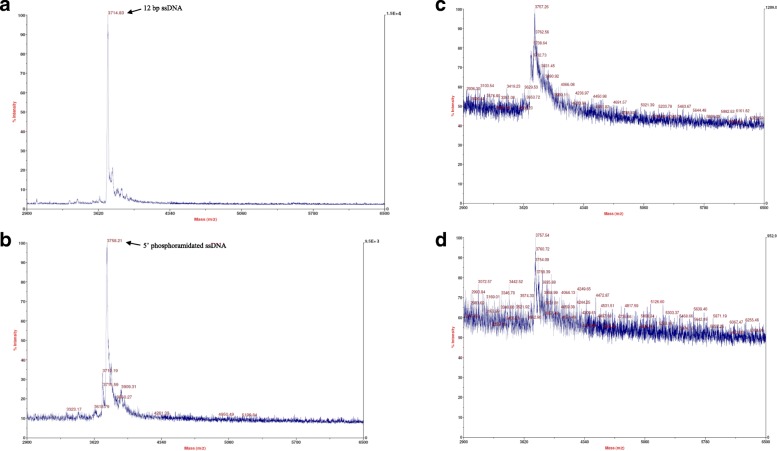
MALDI-TOF spectra of 12 bp ssDNA (**a**), the phosphoramidated conjugate on day 1 (**b**). The phosphoramidated conjugate after 7 days (**c**) and 14 days (**d**) upon storage at − 20 °C (*n* = 5)

MALDI-TOF indicated that a phosphoramidated conjugate is present, and that it would have prolonged usage (*n* = 5). The conjugate signal at 3758 m/z is observed upon storage at − 20 °C (Fig. 3b,c,d). The major peak corresponding to the phosphoramidated ssDNA conjugate is still present even after 14 days, indicating that the product remains intact. As covalent bonds are stronger, these MALDI-TOF results suggest that the formed phosphoramidated ssDNA conjugate product consists of a stable covalent bond, as we observed its presence after longer term storage. However, a decrease in the signal intensity (i.e., increasing noise) is observed upon storage at − 20 °C, which could possibly be due to hydrolysis.

### Phosphoramidation exclusively at 5’phosphate of ssDNA

**Fig. 4:**
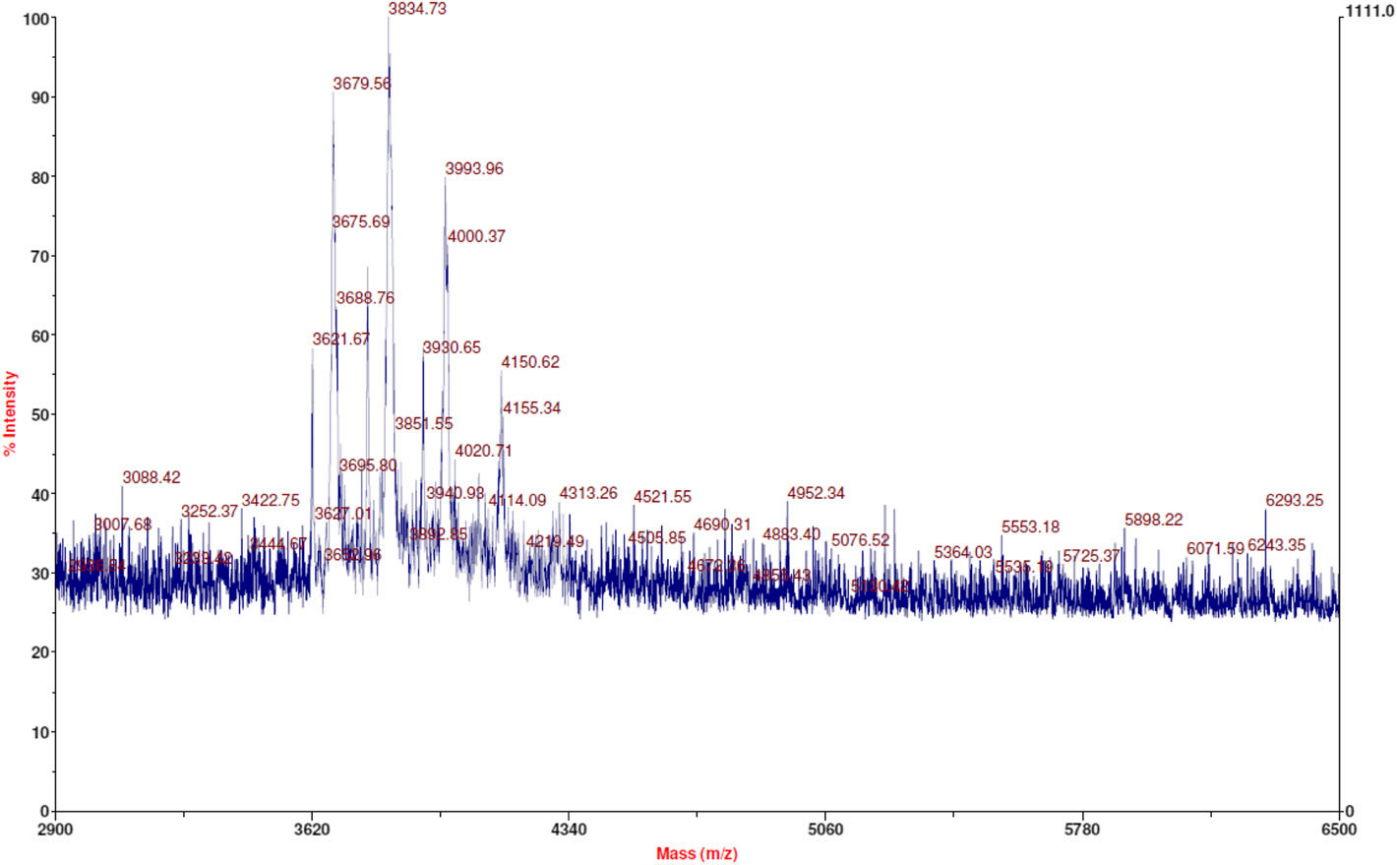
MALDI-TOF spectra indicating multiple peaks attributed to adduct formations. The expected m/z signal at 3758 is not observed in the spectra

As suggested in previous literature, the ssDNA phosphate backbone can form adducts with EDC [18]. These adducts rise from non-covalent, electrostatic interactions between the phosphate groups and EDC. In order to investigate specific covalent bond formation at the open 5′ phosphate of ssDNA, the EDC/Im reaction was conducted with nonphosphorylated ssDNA. As nonphosphorylated ssDNA lacks the open 5′ phosphate group, there should not be any m/z signal owing to the expected phosphoramidated conjugate mass in MALDI-TOF results. As expected, no dominant, single peak was observed around 3758 m/z. Instead, multiple peaks were observed on MALDI-TOF, owing to adducts (Fig. 4). The spectra indicated mass additions to the starting reactant (5′ non phosphorylated ssDNA) ranging from 40 Da – 400 Da. This observation could be attributed to adduct formation via salt binding to the ssDNA phosphate backbone. It could also be due to temporary electrostatic interactions between the phosphate backbone of ssDNA and EDC and/or other small molecules. When MALDI-TOF runs were carried out with the same sample after 7 days of storage at − 20 °C, the initially detected adduct peaks were not observed. Therefore, it was believed that none of the multiple peaks observed initially were due to stable, covalent bonds. It is validated by the observation that those signals no longer appeared on MALDI-TOF after 7 days, indicating dissociation caused by hydrolysis (Additional file 1).

### RP-HPLC: method validation

#### Linearity

**Fig. 5:**
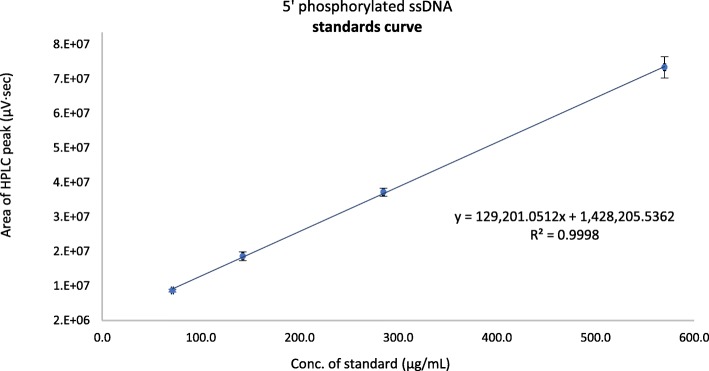
Standards calibration curve of 5′ phosphorylated ssDNA. Column dimensions: XTerra® MS C18 2.5 μm, 4.6 mm × 50 mm. Ion pairing reagent: 0.1 M TEAA, pH 9.70 ± 0.05. Mobile phases: A; 5% acetonitrile in TEAA. B; 30% acetonitrile in TEAA. Gradient conditions: 90%A – 60%A in 16 min. 60–90% A in the next 14 min. Flowrate: 1.0 mL/min. Column and sample temperatures: 37 °C, and 33 °C. Injection volume: 100 μL. UV detector: 260 nm. Error bars represent ±1 SD of the peak areas from triplicate results

The calibration curve was constructed by linear regression using four standards of known concentrations (Fig. 5). The coefficient of determination (r^2^) of the calibration curve was 0.9988. The linear regression equation was; y = 129,201·x + 1,428,205, where; y = area under the HPLC peak (μV·sec), and x = concentration of the analyte (μg/mL).

#### Accuracy, precision, detection limit, and quantitation limit

The accuracy and precision of the developed method are depicted from the %bias and the %CV respectively (Table 2). The %CV for all levels were well below 3%, and the %bias ranged from − 5.1 – 1.2%. These are in agreement with a previously established acceptance criteria in a chromatographic study [21]. The detection limit (LOD) and quantitation limit (LOQ) determined as per the ICH Harmonized Tripartite Guidelines were 4.6 μg/mL and 13.9 μg/mL respectively. All the detected concentrations of the phosphoramidated ssDNA conjugate products were > 278 μg/mL. They are well above the LOQ, and are well within the linear range of the analytical method.

**Table 2:** Recovery analysis and system suitability parameters (*n* = 12 total determinations)

Nominal concentration (μg/mL)	Calculated concentration (μg/mL)	%Recovery	SD (±μg/mL)	%CV	%bias
570.0	568.3	99.7%	1.1	0.2%	−0.3%
285.0	288.3	100.0%	3.7	1.3%	1.2%
142.5	144.5	100.0%	3.3	2.3%	1.4%
71.3	67.6	94.9%	0.8	1.2%	−5.1%

### Phosphoramidated ssDNA conjugate quantitation

**Fig. 6:**
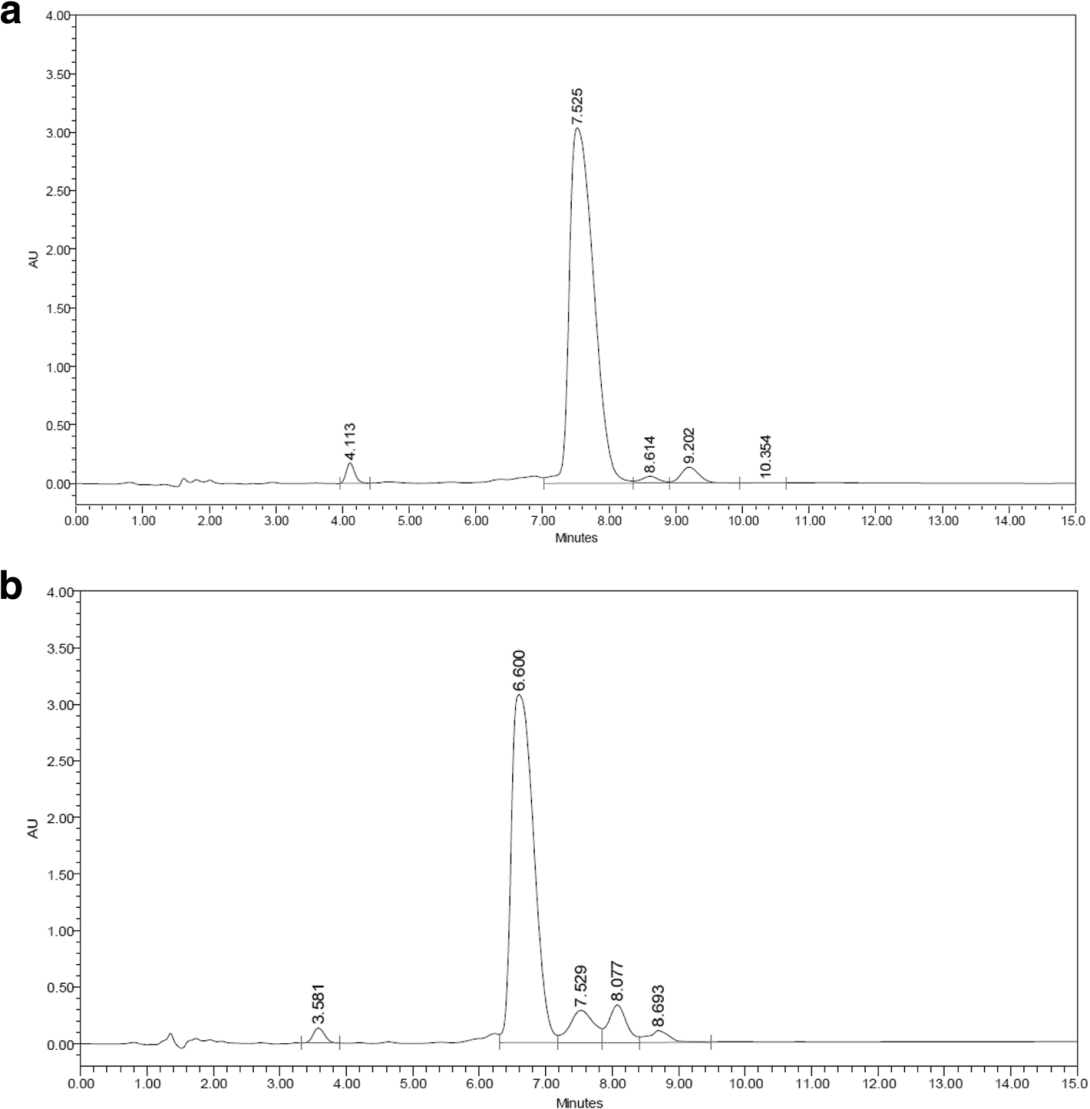
Chromatographic results of the reactant ssDNA (**a**), and the phosphoramidated ssDNA conjugate (**b**). Column dimensions: XTerra® MS C18 2.5 μm, 4.6 mm × 50 mm. Ion pairing reagent: 0.1 M TEAA, pH 9.70 ± 0.05. Mobile phases: A; 5% acetonitrile in TEAA. B; 30% acetonitrile in TEAA. Gradient conditions: 90%A – 60%A in 16 min. 60–90% A in the next 14 min. Flowrate: 1.0 mL/min. Column and samples temperatures: 37 °C, and 33 °C. Injection volume: 100 μL. UV detector: 260 nm

After assessing the developed IP-RP-HPLC method for suitability as reported above, the method was used to determine the phosphoramidated ssDNA conjugate yields obtained via the two reactions. The retention time (RT) of the reactant ssDNA was 7.5 ± 0.2 min, while that of the ssDNA conjugate product was 6.1 ± 0.4 min (Fig. 6).

**Fig. 7:**
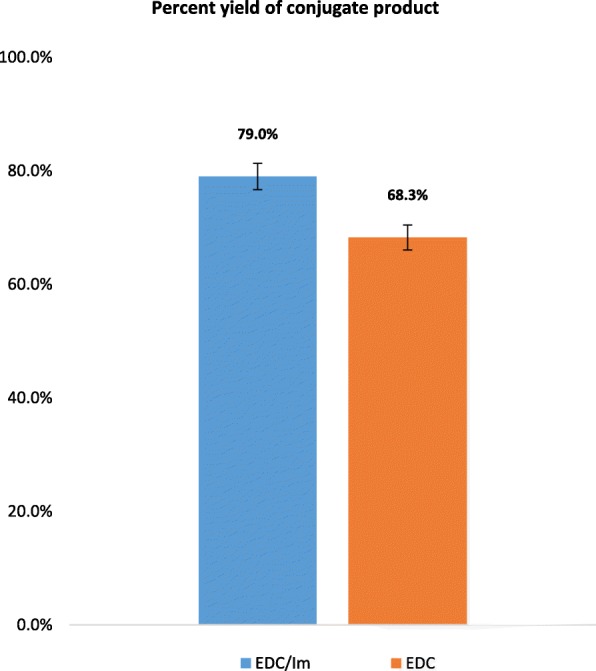
Percent yield of the phosphoramidated ssDNA conjugate via the conventional EDC scheme and the modified EDC/Im reaction strategy (*p* = 0.00008). Error bars represent ±1 SD of the detected product concentrations from n = 5 independent runs per reaction scheme

Figure 7 illustrates the conversion efficiencies obtained via the two separate reaction schemes investigated. Results showed that by following the EDC/Im reaction, the percent yield of the conjugate product increased by approx. 10%. The phosphoramidated ssDNA conjugate percent yield via the EDC/Im reaction method was 79.0 ± 2.4% while that of the EDC reaction method alone was 68.3 ± 2.2%. In order to test whether the observed difference between the conversion efficiencies of the two separate reaction methods was significant, an unpaired, two-tailed, Student’s t-test was conducted. Results indicate that there is a statistically significant difference between the conversion rates (i.e., percent yields) obtained via EDC/Im, and EDC reaction schemes (*p* = 0.00008).

In research studies with multiple crosslinking steps, higher yields of product formation at each step is highly desired. For further yield improvement, additional experimental methods such as increasing the molar amounts of imidazole, and changing reaction conditions (reaction temperature) could be investigated. Another promising method to potentially increase the yield would be to continously mix the reaction during the incubation period.

### Molecular weight analysis of HPLC fractions

In order to determine the molecular composition of the three most dominant HPLC peaks, those fractions were collected as illustrated in Additional file 2, and were analyzed via MALDI-TOF. Results of the major peak (Additional file 2, peak #1) indicated 3764.2 ± 4.2 Da (Additional file 3a). The fractions of one minor peak (Additional file 2, peak #2) showed 3720.9 ± 0.7 Da (Additional file 3b), while the other minor peak (Additional file 2, peak #3) resulted in 3874.6 ± 1.8 Da (Additional file 3c). Peak #1 is attributed to the product conjugate while peak #2 is attributed to unreacted starting material, 5’phosphorylated ssDNA, as the theoretical molecular weights agree with the observed molecular weights.

**Fig. 8:**
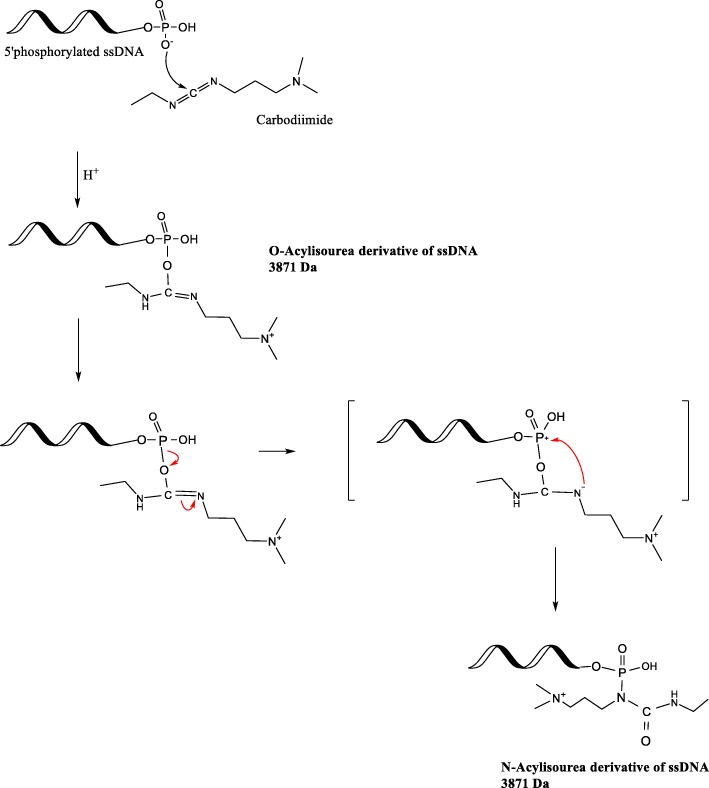
Reaction mechanism deduction for the possible side reactions leading to the N-acylisourea ssDNA byproduct, with a molecular weight of 3871 Da (modified from [1])

In order to determine the molecular structures present in peak #3 eluting at approx. RT = 8.0 min, byproducts possible from EDC side reactions were considered. Other authors have speculated that in the presence of excess EDC, O-acylisourea intermediate could react with the neighboring amine in EDC, forming N-acylisourea [1]. The theoretical molecular weight of both O-acyl isourea, and N-acyl isourea derivatives are 3871 Da (Fig. 8). MALDI-TOF experimental evidence suggests that the molecular composition of the HPLC peak #3 could be due to the presence of acylisourea derivatives (Additional file 3c).

## Conclusions

A novel phosphoramidated ssDNA was synthesized as a proof-of-concept modality to compare the conventional carbodiimide (EDC) reaction with an adapted strategy using EDC and imidazole (EDC/Im). MALDI-TOF results indicated that a stable conjugate with the expected molecular weight was formed. The EDC/Im reaction method was compared to the conventional EDC reaction method in relation to reaction efficiencies. An RP-HPLC chromatographic analytical method was developed to determine the product yields. This method could also be used to purify other aminated and phosphorylated DNA compounds. The phosphoramidated ssDNA conjugate obtained from the EDC/Im method had a percentage yield of 79.0 ± 2.4% while that of the conventional EDC method was 68.3% ± 2.2%. The improved EDC/Im method may be applicable to research related to constructing programmable DNA moieties and constructing self-assembling macromolecules where there is a lack of experimental flexibility in adjusting reaction conditions.

## Additional files

Additional file 1: The adducts formed when unphosphorylated ssDNA was used in the EDC conjugation chemistry. The peaks observed on day 1 has all been deteriorated after 7 days of storage at −20 °C. This indicates that the bonds formed initially were not covalent bonds. The masses greater than the starting material (< 3641 Da) in here are expected to be gas phase dimerization results during MALDI-TOF analyses. (DOCX 102 kb)

Additional file 2: Chromatograph of phosphoramidated conjugate illustrating peaks collected for molecular weight analysis via MALDI-TOF. The fractions were collected for validation of the species in the major peak (#1) as well as the minor peaks (#2, #3). Column dimensions: XTerra® MS C18 2.5 μm, 4.6 mm × 50 mm. Ion pairing reagent: 0.1 M TEAA, pH 9.7 ± 0.05. Mobile phases: A; 5% acetonitrile in TEAA. B; 30% acetonitrile in TEAA. Gradient conditions: 90%A – 60%A in 16 min. 60–90% A in the next 14 min. Flowrate: 1.0 mL/min. Column and sample temperatures: 37 °C, and 33 °C. Injection volume: 100 μL. UV detector: 260 nm. (DOCX 123 kb)

Additional file 3: MALDI-TOF spectra illustrating the m/z values of the HPLC peaks (a) peak #1–phosphoramidated ssDNA. (b) peak #2–unreacted starting ssDNA. (c) peak #3–isourea intermediate derivative (*n* = 3). (DOCX 269 kb)

## Data Availability

The data contained in this submission is freely available for non-commercial purposes. Please contact the corresponding author to obtain data.
