# Supplementary material for: Characterization of covalent crosslinking strategies for synthesizing DNA-based bioconjugates
Source: J Biol Eng. 2019 Jul 10;13:63. doi: 10.1186/s13036-019-0191-2 (PMC6621941; doi:10.1186/s13036-019-0191-2)
Supplement: Supplementary file 1 — The adducts formed when unphosphorylated ssDNA was used in the EDC conjugation chemistry. The peaks observed on day 1 has all been deteriorated after 7 days of storage at −20 °C. This indicates that the bonds formed initially were not covalent bonds. The masses greater than the starting material (< 3641 Da) in here are expected to be gas phase dimerization results during MALDI-TOF analyses. (DOCX 102 kb) [file 13036_2019_191_MOESM1_ESM.docx]

Additional files


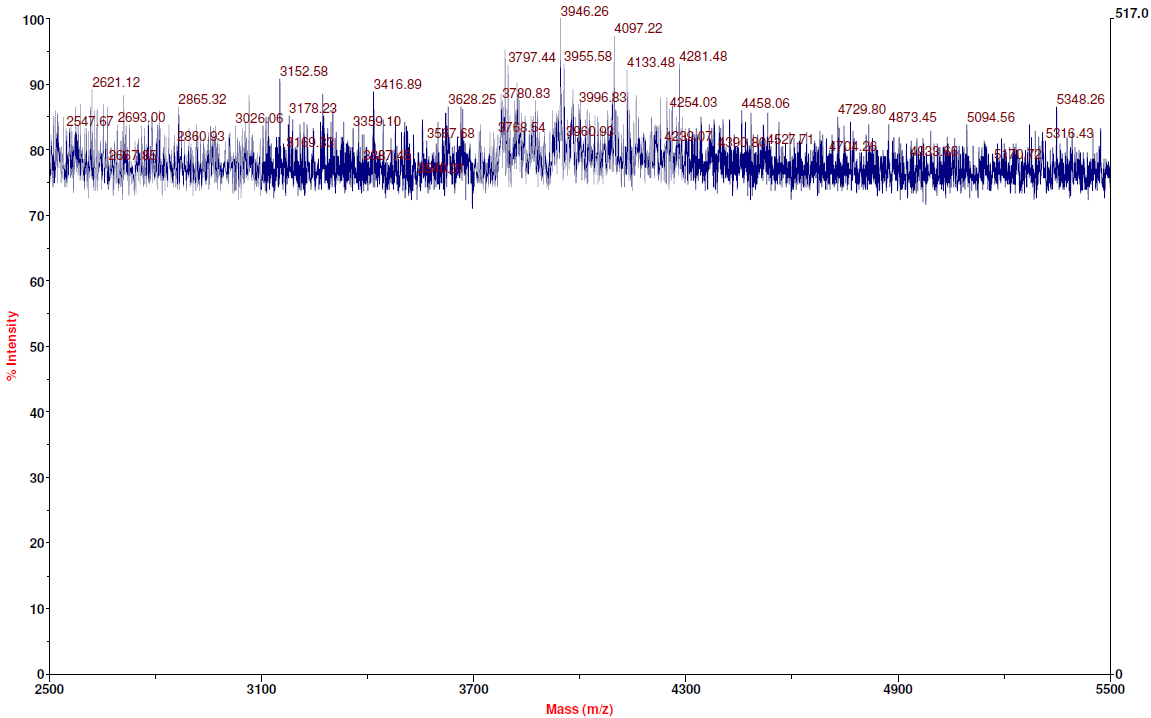


**Figure S1:** The adducts formed when unphosphorylated ssDNA was used in the EDC conjugation chemistry. The multiple peaks observed on day 1 have all been deteriorated after 7 days of storage at -20 °C. This indicates that the bonds formed initially were not covalent bonds. The masses greater than the starting material (<3641 Da) in here are expected to be gas phase dimerization results during MALDI-TOF analyses.
