# Supplementary material for: Characterization of covalent crosslinking strategies for synthesizing DNA-based bioconjugates
Source: J Biol Eng. 2019 Jul 10;13:63. doi: 10.1186/s13036-019-0191-2 (PMC6621941; doi:10.1186/s13036-019-0191-2)
Supplement: Supplementary file 2 — Chromatograph of phosphoramidated conjugate illustrating peaks collected for molecular weight analysis via MALDI-TOF. The fractions were collected for validation of the species in the major peak (#1) as well as the minor peaks (#2, #3). Column dimensions: XTerra® MS C18 2.5 μm, 4.6 mm × 50 mm. Ion pairing reagent: 0.1 M TEAA, pH 9.7 ± 0.05. Mobile phases: A; 5% acetonitrile in TEAA. B; 30% acetonitrile in TEAA. Gradient conditions: 90%A – 60%A in 16 min. 60–90% A in the next 14 min. Flowrate: 1.0 mL/min. Column and sample temperatures: 37 °C, and 33 °C. Injection volume: 100 μL. UV detector: 260 nm. (DOCX 123 kb) [file 13036_2019_191_MOESM2_ESM.docx]

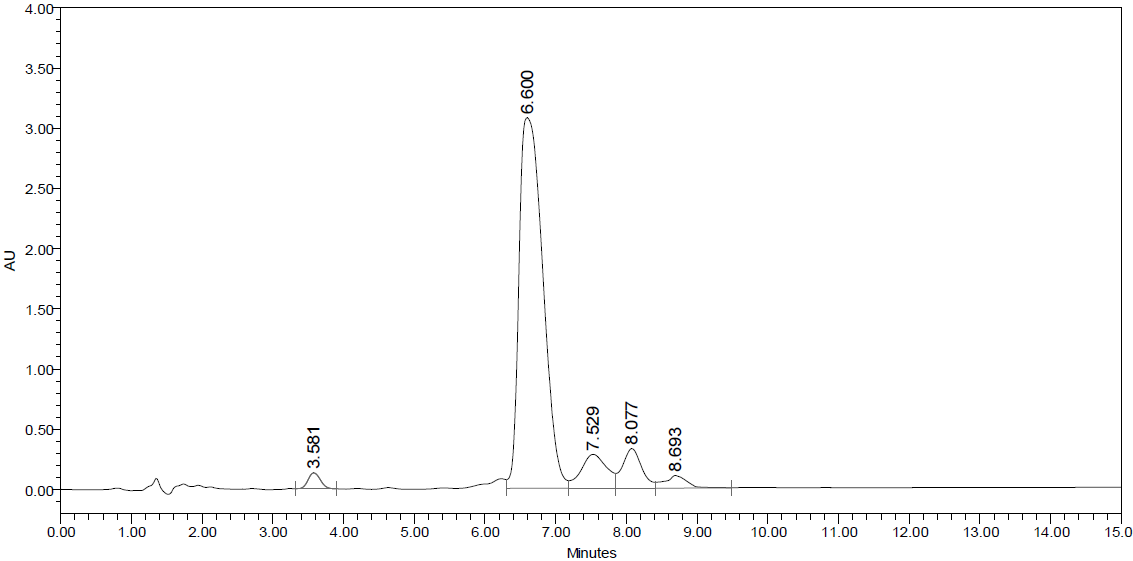


**#1**

**#2**

**#3**

**Figure S2**: Chromatograph of phosphoramidated conjugate illustrating peaks collected for molecular weight analysis via MALDI-TOF. The fractions were collected for validation of the species in the major peak (#1) as well as the minor peaks (#2, #3). Column dimensions: XTerra^®^ MS C18 2.5 µm, 4.6 mm × 50 mm. Ion pairing reagent: 0.1 M TEAA, pH 9.7±0.05. Mobile phases: A; 5% acetonitrile in TEAA. B; 30% acetonitrile in TEAA. Gradient conditions: 90%A – 60%A in 16 min. 60% – 90% A in the next 14 min. Flowrate: 1.0 mL/min. Column and sample temperatures: 37°C, and 33°C. Injection volume: 100 µL. UV detector: 260 nm.
