# Supplementary material for: Characterization of covalent crosslinking strategies for synthesizing DNA-based bioconjugates
Source: J Biol Eng. 2019 Jul 10;13:63. doi: 10.1186/s13036-019-0191-2 (PMC6621941; doi:10.1186/s13036-019-0191-2)
Supplement: Supplementary file 3 — MALDI-TOF spectra illustrating the m/z values of the HPLC peaks (a) peak #1–phosphoramidated ssDNA. (b) peak #2–unreacted starting ssDNA. (c) peak #3–isourea intermediate derivative (n = 3). (DOCX 269 kb) [file 13036_2019_191_MOESM3_ESM.docx]

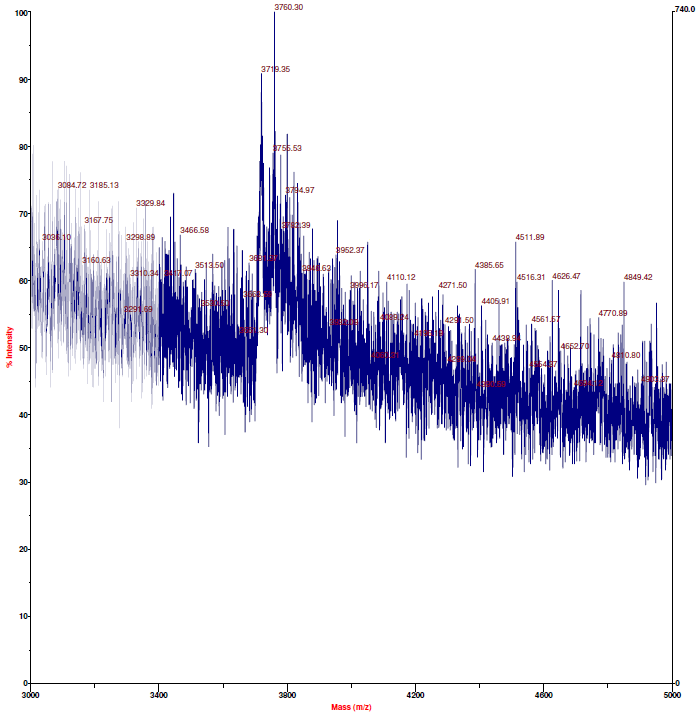


(a)


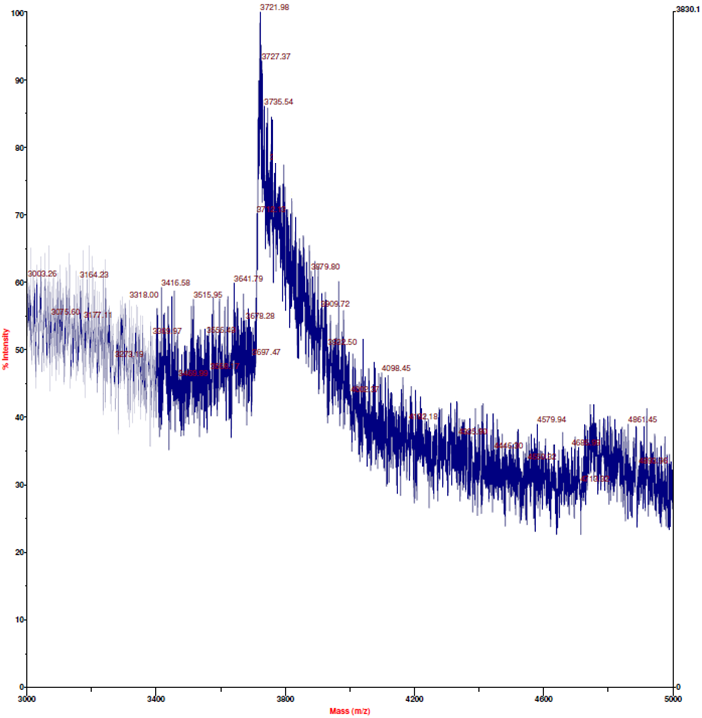


(b)


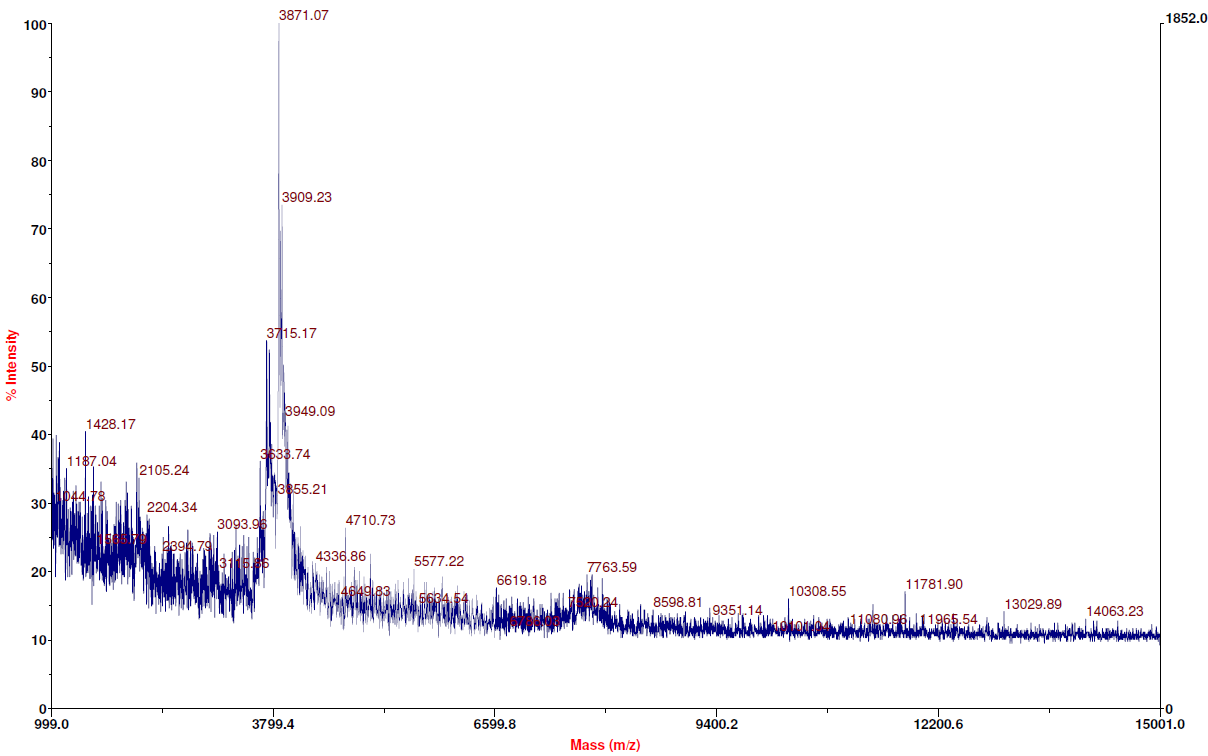


(c)

**Figure S3**: MALDI-TOF spectra illustrating the m/z values of the HPLC peaks **(a)** peak #1–phosphoramidated ssDNA. **(b)** peak #2–unreacted starting ssDNA. **(c)** peak #3–isourea intermediate derivative (n=3).
